# Tumor-treating fields elicit a conditional vulnerability to ionizing radiation via the downregulation of BRCA1 signaling and reduced DNA double-strand break repair capacity in non-small cell lung cancer cell lines

**DOI:** 10.1038/cddis.2017.136

**Published:** 2017-03-30

**Authors:** Narasimha Kumar Karanam, Kalayarasan Srinivasan, Lianghao Ding, Brock Sishc, Debabrata Saha, Michael D Story

**Affiliations:** 1 Division of Molecular Radiation Biology, Department of Radiation Oncology, University of Texas Southwestern Medical Center, Dallas, TX, USA; 2 Simmons Comprehensive Cancer Center, University of Texas Southwestern Medical Center, Dallas, TX, USA

## Abstract

The use of tumor-treating fields (TTFields) has revolutionized the treatment of recurrent and newly diagnosed glioblastoma (GBM). TTFields are low-intensity, intermediate frequency, alternating electric fields that are applied to tumor regions and cells using non-invasive arrays. The predominant mechanism by which TTFields are thought to kill tumor cells is the disruption of mitosis. Using five non-small cell lung cancer (NSCLC) cell lines we found that there is a variable response in cell proliferation and cell killing between these NSCLC cell lines that was independent of p53 status. TTFields treatment increased the G2/M population, with a concomitant reduction in S-phase cells followed by the appearance of a sub-G1 population indicative of apoptosis. Temporal changes in gene expression during TTFields exposure was evaluated to identify molecular signaling changes underlying the differential TTFields response. The most differentially expressed genes were associated with the cell cycle and cell proliferation pathways. However, the expression of genes found within the BRCA1 DNA-damage response were significantly downregulated (*P*<0.05) during TTFields treatment. DNA double-strand break (DSB) repair foci increased when cells were exposed to TTFields as did the appearance of chromatid-type aberrations, suggesting an interphase mechanism responsible for cell death involving DNA repair. Exposing cells to TTFields immediately following ionizing radiation resulted in increased chromatid aberrations and a reduced capacity to repair DNA DSBs, which were likely responsible for at least a portion of the enhanced cell killing seen with the combination. These findings suggest that TTFields induce a state of ‘BRCAness' leading to a conditional susceptibility resulting in enhanced sensitivity to ionizing radiation and provides a strong rationale for the use of TTFields as a combined modality therapy with radiation or other DNA-damaging agents.

Lung cancer is the second most prevalent cancer and the leading cause of cancer-related death in the United States.^1^ Non-small cell lung cancer (NSCLC) is the most prevalent type, accounting for ~80% of new cases.^2, 3^ A plethora of treatment options exist including surgical resection, chemotherapy, radiation therapy and immunotherapy.^4, 5^ Five-year survival rates for patients with stage I and II NSCLC are ~50% and 30%, respectively. However, despite this myriad of options, 5-year survival rates for patients with late stage IIIA, IIIB and IV are 14%, 5% and 1%, respectively (www.cancer.net), highlighting the need for novel treatment modalities that can be utilized alone or in combination with conventional therapies to increase survival rates.

The advent of Tumor-Treating Fields (TTFields), a novel physical treatment modality, has been effective for the treatment of solid, therapy-resistant primary and recurrent tumors.^6, 7, 8, 9^ TTFields electrodes are non-invasive and deliver a low-intensity (1–3 V/cm) intermediate frequency (100–300 kHz) alternating electric field across the tumor bed.^10^ TTFields create a heterogeneous intracellular environment that induces a dielectrophoretic movement of polar molecules toward the region of higher field intensity, effectively preventing polymerization and other critical biochemical functions.^11^ As such, TTFields preferentially target cancer cells through the exploitation of cell proliferation, effectively sparing non-dividing normal cells. In addition, TTFields do not stimulate nerves and muscle because of their high frequency, and do not generate heat because of their low intensity.^10^ The FDA has approved Optune (NovoCure), a TTFields generating transducer array, for the treatment of recurrent and newly diagnosed glioblastoma (GBM) in combination with temozolomide.^12^ Clinical trials are ongoing or recruiting for cancers at additional anatomic sites including lung, pancreatic and ovarian cancers (www.novocure.com).

TTFields are known to decrease cellular proliferation and induce abortive apoptosis in dividing cancer cells across a variety of human and rodent tumor cell lines.^13^ Prevention of proper formation of the mitotic spindle apparatus and the activation of the mitotic spindle checkpoint has been proposed as the mechanism by which TTFields kill dividing cells.^14, 15^ Specifically, TTFields exposure leads to microtubule depolymerization and the mislocalization of septin. This results in plasma membrane instability and blebbing that disrupts cytokinesis, leading to abnormal chromosome segregation, aberrant mitotic exit and production of deranged cells that subsequently undergo apoptosis.^16^ In the context of cancer therapy, TTFields has been shown to enhance the efficacy of numerous chemotherapeutic agents when used in combination such as paclitaxel and doxorubicin in multidrug-resistant cancer cells—without increasing the intracellular accumulation of the drugs;^17^ decreased cellular proliferation, survival and the percentage of G2/M populations; enhanced the efficacy of chemotherapy in a hamster pancreatic cancer model;^6^ decreased cellular proliferation and enhanced the efficacy of pemetrexed, cisplatin and paclitaxel in NSCLC cells both *in vitro* and *in vivo,*^18^ all lending support for the use of TTFields in a combination setting.^19^ In addition to its efficacy in treating primary tumors, TTFields have also demonstrated the ability to prevent or delay metastasis in animal models, a process likely resulting from enhancement of the antitumor immune response.^20^ Furthermore, it was recently shown that TTFields enhanced the efficacy of radiation treatment through the induction of increased mitotic abnormalities and induction of DNA damage in GBM.^21^ Although these findings collectively support the efficacy of TTFields as an anticancer agent, further mechanistic insights are needed to optimize the use of TTFields in combination with additional modalities including radiation therapy.

Using a panel of NSCLC cell lines with different molecular phenotypes, we found that TTFields alone exhibits antiproliferative effects and cytotoxicity. We divided NSCLC cells into more responsive (H157 and H4006) and less responsive (A549, H129, H1650) cell lines based on their degree of responsiveness to TTFields. Consistent with previous reports, we also observed a time-dependent increase in the G2/M population upon TTFields exposure; however, the number of cells accumulating in G2/M was likely not enough to account for the decreased survival seen. Therefore, we postulated that TTFields may induce additional mechanisms leading to cell death. To explore this phenomenon and identify novel mechanisms that could be exploited clinically, we performed temporal gene expression analysis after treating H157, H4006, A549, H1650 and H1299 cell lines with TTFields for up to 48 h. In addition to confirming previously described mechanisms with the perturbation of genes involved in cell cycle regulation and mitosis, we also identified a significant association of differentially expressed genes to the BRCA1 pathway (*P*<0.05) upon TTFields treatment. This finding suggests that a novel mechanism involving DNA repair and/or DNA replication may contribute to TTFields induced cell killing other than the reported abortive mitosis cell death mechanism. TTFields alone elevated the frequency of chromatid-type aberrations and induced *γ*-H2AX foci, in addition to slowing the repair of ionizing radiation (IR)-induced double-strand breaks (DSBs). As expected, TTFields sensitized NSCLC cells to IR and decreased the surviving fraction at 2 and 4 Gy. The effect was at least additive and, in some cases, synergistic. Taken together, these results highlight a previously unknown mechanism for TTFields-induced cell killing, and also suggest that TTFields may establish a ‘conditional vulnerability' resulting from an induced state of ‘BRCAness' effectively sensitizing cells to IR and opening new avenues for combination therapy with DNA-damaging agents and other agents such as PARP inhibitors.

## Results

### TTFields reduce NSCLC cell proliferation

Previous studies have demonstrated that TTFields exhibit optimal effectiveness in a cell line-specific manner.^6, 18, 22^ To determine the optimal frequency to maximize growth inhibition, cells were treated at different frequencies ranging from 100 to 300 kHz. Cell counts were taken every 24 h for up to 72 h within a panel of NSCLC cell lines (Supplementary Figure 1). The list of cell lines utilized in this study, their standardized TTFields frequencies, cell-doubling times, percentage of growth inhibition at their optimized frequency at 72 h and their p53 and KRAS mutation status are listed in Table 1. Further experimentation was carried out at the optimal frequencies listed in this table. While TTFields reduced the growth in all the cell lines examined, its relative efficacy was lower in the H1650, H1299 and A549 cell lines and higher in the H157 and H4006 cell lines (Table 1).

### TTFields exposure causes cell death in NSCLC cell lines

**Figure 1:**
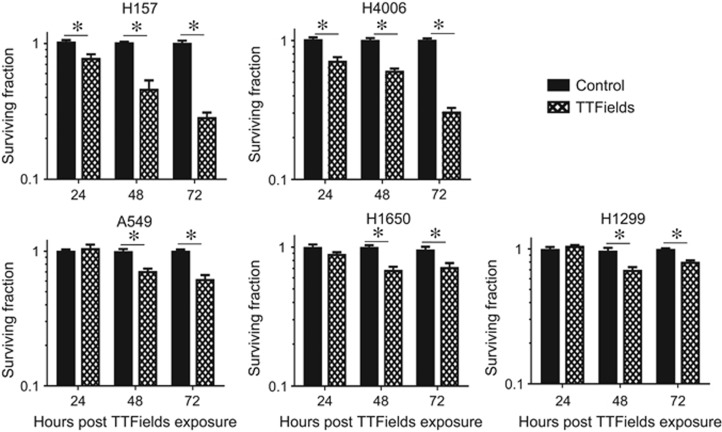
TTFields treatment induces NSCLC cell death. The fraction of cells surviving TTFields treatment at 24, 48 and 72 h post induction in a panel of NSCLC cell lines including H157, H1299, A549, H1650 and H4006. Values are represented as the number of colony-forming cells relative to control. Error bars represent the S.E.M. of three separate experiments and asterisks represent values where survival was significantly (*P*<0.05) decreased

As growth delay is not the same as cell killing, we examined the ability of TTFields to induce reproductive cell death using clonogenic survival assays. TTFields treatment resulted in a significant decrease in cell survival in all the cell lines examined, a trend that generally increased with the amount of time cells were exposed to TTFields. As with the cell growth patterns, H1650, H1299 and A549 were less responsive and H157 and H4006 more responsive, to TTFields (Figure 1). The characterization of more responsive *versus* less responsive was maintained for all assays in this study.

### TTFields exposure alters the cell cycle distribution by enriching the G2/M population and generating a sub-G1 population

**Figure 2:**
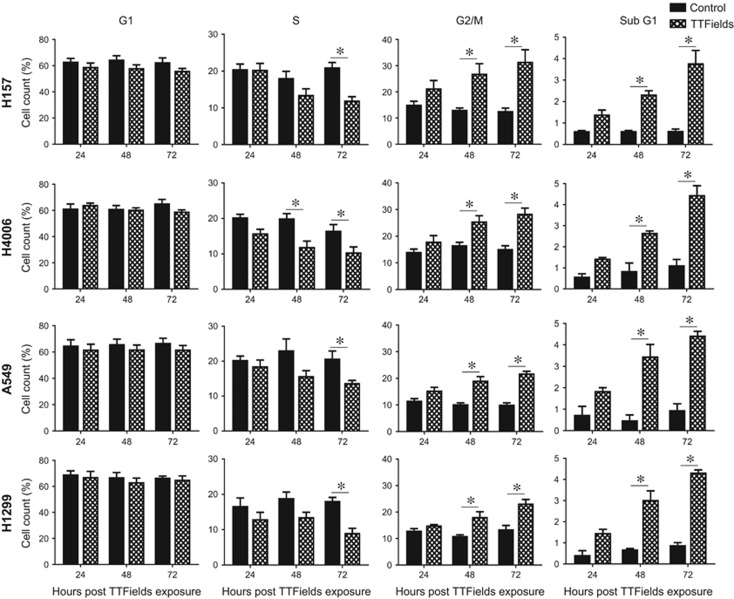
TTFields alter cell cycle distribution. TTFields treatment resulted in a significant enrichment of NSCLC cells in the G2/M phase of the cell cycle, a decrease in the percentage of cells in S-phase, and resulted in the significant induction of a sub-G1 population indicative of an apoptotic cell population. Error bars represent the S.E.M. of three separate experiments and asterisks represent significant changes (*P*<0.05) in cell count percentage at given time point and cell line

Previous reports have established that TTFields alter the cell cycle distribution, resulting in an increase in the G2/M phase of the cell cycle with increasing treatment time in GBM and ovarian cancer cell lines.^13, 22^ To determine whether TTFields induce a similar enrichment of the G2/M population in NSCLC cell lines, we performed propidium iodide (PI) staining and examined the distribution of cells throughout the cell cycle using flow cytometry in the two most responsive cell lines (H157 and H4006) and two of the less responsive cell lines (A549 and H1299). Consistent with previous reports TTFields treatment enriched the G2/M and G0/G1 populations while decreasing the number of S-phase cells in all cell lines tested (Figure 2). Furthermore, TTFields generated a sub-G1 population, giving strong, albeit not definitive, evidence for an apoptotic cell population. Representative histograms are shown in Supplementary Figure 2. These changes in cell cycle distribution are likely not sufficient to account for the amount of cell death observed with TTFields application. Therefore, we postulated that additional mechanism(s), aside from cell cycle perturbation and abortive apoptosis following mitosis, must be contributing to TTFields-induced cell death.

### TTFields induce global gene expression changes

**Figure 3:**
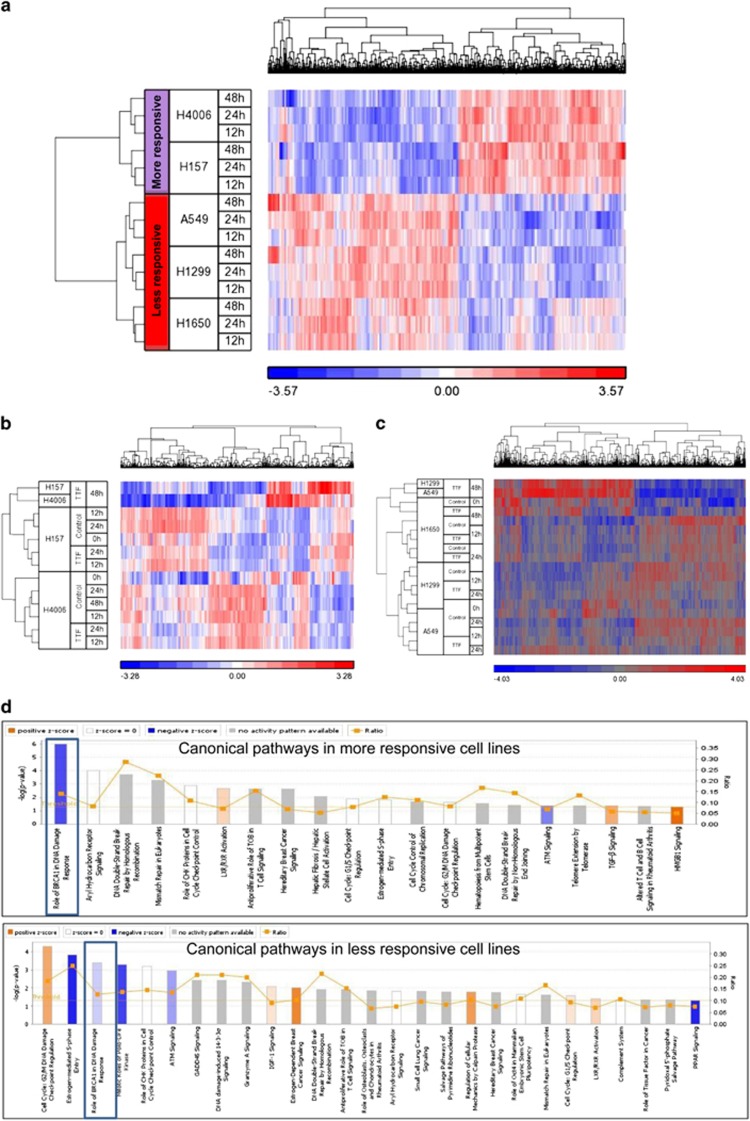
TTFields treatment induces gene expression changes and downregulates BRCA1 DNA damage repair pathway. (**a**) Supervised clustering using a 1083 gene (FDR<0.01) signature that segregates cell lines by response to TTFields exposure. The heatmap scale is based upon log2 values of normalized gene expression. Clustering analysis of differentially expressed genes after TTFields treatment revealed that (**b**) 628 genes (FDR<0.05) responded to TTFields in the more responsive cell lines and (**c**) 1039 genes (FDR<0.05) responded to TTFields in the less responsive cell lines. (**d**) Identification of differentially regulated canonical pathways for TTFields exposure in the more responsive and the less responsive cell lines. Downregulation of the BRCA1 DNA damage repair pathway is more pronounced in the more responsive cell lines compared to the less responsive cell lines, which is denoted by a decrease in the intensity of blue color reflecting the negative *z*-scores

To explore alternative potential mechanisms for TTFields-induced cell death, we performed gene expression analysis on our panel of NSCLC cells exposed to TTFields for up to 48 h. A schematic of experimental time points and cell lines is given in Supplementary Figure 3. Differential gene expression after TTFields exposure was examined using significance analysis of microarray (SAM) time course analysis. By normalizing TTFields-induced gene expression to the baseline gene expression values for each cell line, we identified a 1083 gene (false discovery rate (FDR)<0.01) signature that segregates cell lines by response to TTFields exposure (Figure 3a). Gene expression analysis was subsequently performed in more responsive and less responsive cell groups, respectively. The analysis suggested that, as a result of TTFields exposure, the expression of 1039 genes was altered in the less responsive cell lines and that 628 genes were differentially expressed in the more responsive NSCLC cell lines. Cluster analysis showed distinct expression profiles that separated the 48 h time point from earlier time points relative to untreated controls in the more responsive lines as well as in less responsive cell lines (Figures 3b and c).

Ingenuity pathway analysis (IPA) was performed to determine specific canonical pathways involved in the TTFields responding genes (Figure 3d). The results suggested that alterations occurred in cell cycle and mitotic regulatory pathways, which is consistent with previous studies but also revealed a significantly downregulated BRCA1 DNA-damage response pathway (*P*<0.05) with TTFields exposure.

### BRCA1 pathway genes are downregulated as a result of TTFields treatment

**Figure 4:**
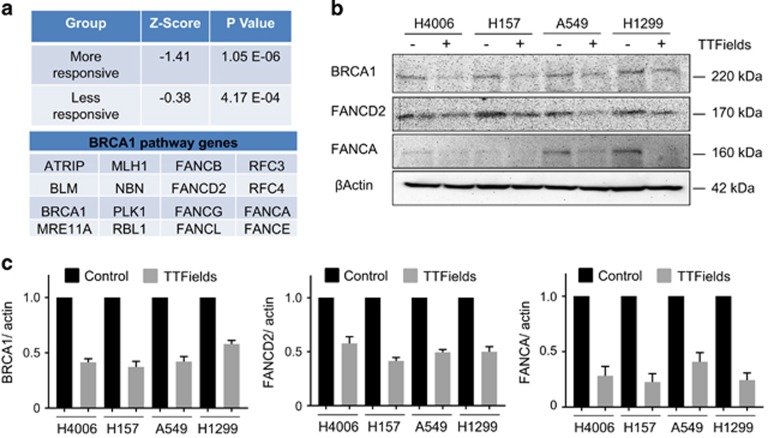
The BRCA1 pathway is downregulated in NSCLC cells exposed to TTFields. (**a**) *z*-scores and *P*-values of BRCA1 pathway gene expression along with the relevant pathway gene names. (**b**) Immunoblot of representative BRCA1 pathway genes demonstrating the downregulation of BRCA1, FANCD2 and FANCA protein levels resulting from TTFields treatment at 72 h. (**c**) Quantification of BRCA1, FANCD2 and FANCA protein levels from immunoblots (*N*=3)

IPA analysis of differentially expressed genes suggested that the activity of the BRCA1 pathway was inhibited as a result of TTFields exposure. Although inhibition occurred in both groups, we observed a stronger inhibition in the more responsive cell lines compared to the less responsive cell lines as indicated by the negative *z*-scores (Figure 4a). Temporal gene expression graphs showing downregulation of many of the BRCA1 pathway genes in all cell lines is found in the Supplementary Figure 4. The confirmation of BRCA1 pathway gene downregulation at the protein level was conducted with immunoblotting for BRCA1, FANCD2 and FANCA (Figure 4b). The quantification of immunoblots revealed that BRCA1, FANCD2 and FANCA protein levels were significantly downregulated in all cell lines at 72 h post-TTFields exposure, confirming gene expression results (Figure 4c).

### TTFields cause DNA damage, reduce IR-induced DNA repair and increase the frequency of chromatid-type aberrations

**Figure 5:**
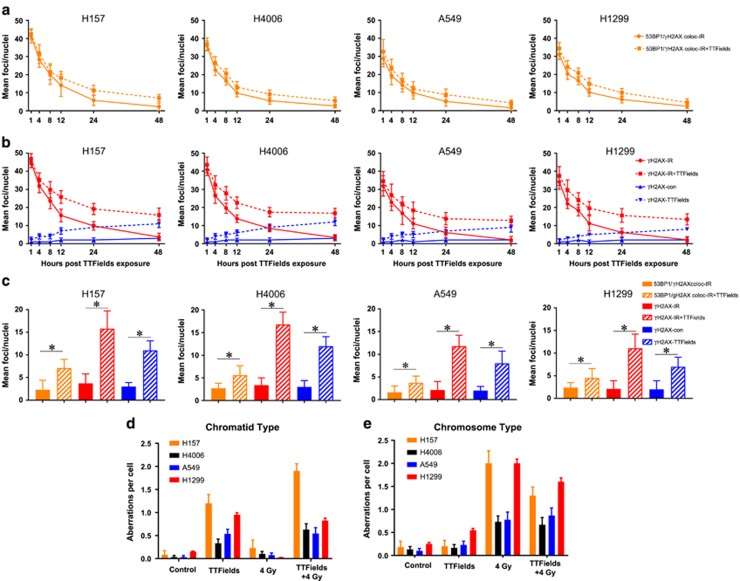
TTFields induce DNA damage and slow the repair of IR-induced DSBs. (**a**) Changes in the mean number of localized 53BP1 and *γ*-H2AX foci over 48 h. (**b**) Change in the mean number of *γ*-H2AX foci over time with TTFields alone (blue lines) and after receiving 2 Gy (red lines), both followed over 48 h. (**c**) The mean value for residual *γ*-H2AX foci and localized 53BP1 and *γ*-H2AX foci at 48 h post-IR for all cell lines. (**d**) TTFields exposure for 48 h resulted in the induction of chromatid-type aberrations in the panel of NSCLC lines. (**e**) The frequency of chromosome-type aberrations after a 48 h TTFields exposure in combination with radiation. Error bars represent the S.E.M. of three separate experiments and asterisks represent significant difference (*P*<0.05) between indicated conditions

Because TTFields decreased BRCA1-associated gene expression, we wanted to confirm whether this resulted in DNA damage induction as a result of TTFields exposure alone or whether there would be a reduction in DNA DSB repair kinetics after IR. Exposure to TTFields alone resulted in the formation of *γ*-H2AX foci, with the mean number of foci per cell increasing as a function of time, indicating that TTFields treatment alone is capable of causing DNA damage (Figures 5b and c). An IR exposure of 2 Gy immediately followed by TTFields application decreased the resolution of *γ*-H2AX foci and colocalized *γ*-H2AX/53BP1 foci, indicating that in addition to causing DNA damage TTFields also reduced the repair of IR-induced damage (Figures 5a and b). When residual lesions at 24 and 48 h were compared, the more responsive cell lines had greater numbers of residual lesions compared to those cell lines considered as less responsive (Figures 5b and c). Representative immunofluorescence images are found in Supplementary Figure 5.

To further confirm the effects of TTFields on DNA damage, we performed cytogenetic analysis to validate the findings associated with DNA repair foci at 48 h post irradiation. TTFields alone significantly increased the frequency of chromatid-type but not chromosome-type aberrations (Figures 5d and e) in all cell lines examined, consistent with the finding that TTFields cause DNA damage. Representative chromosome spreads were shown in Supplementary Figure 6. The combined effect of TTFields plus IR increased both chromatid-type and chromosome-type aberrations, albeit not at a higher frequency than that of each agent alone.

### TTFields sensitize NSCLC cells to IR

**Figure 6:**
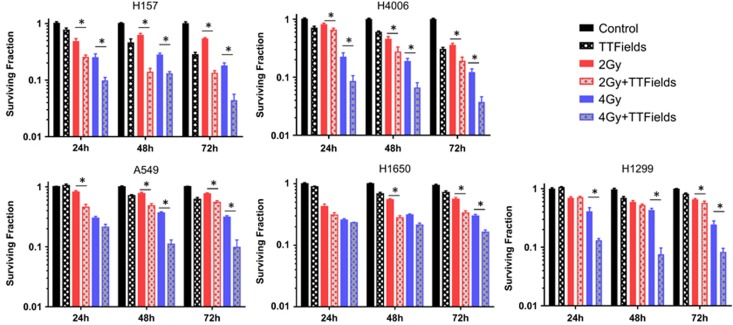
TTFields sensitize NSCLC cells to IR. TTFields treatment was applied alone or immediately following treatment with 2 or 4 Gy of ^137^Cs *γ*-rays. Survival was then assessed in all cell lines following 24, 48 or 72 h of TTFields induction. Error bars represent the S.E.M. of three separate experiments and asterisks represent values where survival was significantly decreased (*P*<0.05; Table 2)

After observing the reduction in BRCA1 expression, a reduction in DNA DSB repair capacity and increased chromatid damage with TTFields exposure, the radioresponse of our panel of NSCLC cell lines was determined via clonogenic cell survival after the cells received either 2 or 4 Gy IR followed by TTFields treatment for 24, 48 and 72 h. All of the cell lines displayed an enhanced sensitivity to IR, although, consistent with earlier results, the degree of sensitization varied between cell lines (Figure 6). We considered the combined effect of TTFields and IR to be synergistic if the combination index (CI) was >1 and the *P*-value was<0.05; (see Materials and Methods). The combined effect of 4 Gy IR and TTFields on cell death was found to be synergistic in all the cell lines tested, whereas the combined effect of 2 Gy IR and TTFields on cell death was found to be synergistic in the H157, H4006, A549 and H1650 cell lines, which is denoted by bold text in Table 2.

## Discussion

In agreement with previous reports,^13, 21, 22^ we confirmed that TTFields have antiproliferative effects, induce cell death and alter the distribution of cells through the cell cycle, resulting in an enrichment of G2/M populations and the generation of a sub-G1 population indicative of apoptotic cells.

Earlier, Gera *et al.*^16^ showed that TTFields sensitivity is dependent on p53 status in colon cancer cells; however, cell proliferation and survival results from our study and recent studies by others^13, 22^ suggest that TTFields effects are independent of p53 status (Table 1 and Figure 1). Because the presence of a sub-G1 population and the increase in G2/M cells are likely not sufficient to account for the differences in survival observed when TTFields were applied across our NSCLC cell panel, we postulated that there are other novel mechanism(s) by which TTFields lead to cell killing. We divided the NSCLC cell lines into two categories, that is, more responsive cell lines (H157 and H4006) and less responsive (A549, H1299 and H1650) cell lines, and conducted gene expression analysis to understand the basis for the differential response of NSCLC cell lines to TTFields.

The molecular basis of the differential responses to TTFields was demonstrated by supervised clustering analysis that clearly segregated the cell lines into a more responsive cluster and a less responsive cluster (Figure 3a). To further elucidate the differences, we compared signaling pathways involved in the genes that responded to TTFields in each of the two cell line groups (Figures 3b and c). The majority of the pathways were common in more responsive (15 out of 19 associated pathways) and less responsive cell lines (15 out of 27 associated pathways), which are related to cell cycle and DNA-damage response pathways (Figure 3d). While these pathways have been reported in previous studies, downregulation of the BRCA1 signaling pathway with TTFields exposure is a novel finding. The fact that BRCA1 pathway downregulation is more pronounced in the more responsive cell lines than in the less responsive cell lines, evident by the negative *z*-scores (Figure 4a), suggests an inverted correlation between BRCA1 pathway activity and the sensitivity of cellular response to TTFields.

BRCA1 together with BRCA2 have an important role in maintaining replication fidelity through the repair of DSB damage by mediating homologous recombination and through non-homologous end joining during S and G2 phases of cell cycle.^23^ DSBs can occur during IR exposure or as by-products of DNA replication. BRCA1 mutant mice exhibit chromosome translocation and chromatid aberrations,^24^ and BRCA2 mutant mice accumulate chromatid breaks and aberrant chromatid exchanges.^25^ BRCA1 defects have been identified in multiple cancers including breast and pancreas.^26, 27^ Defects in the BRCA genes predispose cells to therapeutics targeting single-strand break (SSB) repair pathways, such as PARP inhibitors, resulting in what has been coined ‘synthetic lethality'.^28^ On the basis of our findings, we propose that TTFields exposure induces a conditional vulnerability, that is, they induce BRCAness^29^ because of the downregulation of the BRCA1 pathway genes. If this is accurate, then TTFields could be applied in combination with PARP inhibitors without the potential for developing therapy-resistant recurrent tumors as is common with molecularly targeted therapies. This is supported by our results where we saw the gradual accumulation of *γ*-H2AX foci following TTFields application over time and slowed DNA repair kinetics following IR exposure (*γ*-H2AX foci and colocalized *γ*-H2AX and 53BP1 foci (Figures 5a–c). Indeed, the more responsive cell lines had, on average, more residual DNA repair foci at 24 and 48 h post-IR than the less responsive cell lines, and Kim *et al*. ^21^ also showed accumulation of *γ*-H2AX upon TTFields treatment but without providing any mechanistic details behind their observation. 53BP1 localizes to DNA DSBs,^30, 31^ which are physically distinct from DNA replication stress,^32, 33^ whereas *γ*-H2AX recruits MRE11, KU70, KU80 and RAD51 to stalled replication forks at early time points.^34^ Therefore, we believe that TTFields not only slow down DNA damage repair kinetics but also induce replication stress based upon the significant differences seen in colocalized *γ*-H2AX/ 53BP1 foci (Figures 5a and c) and *γ*-H2AX foci alone (Figures 5b and c). Furthermore, the increased frequency of chromatid-type aberrations (Figures 5d and 5e) is consistent with ongoing replicative stress induced by TTFields as it is known that a defective response to replication stress leads to an accumulation of chromatid-type aberrations.^35, 36^ Hence, we postulate that TTFields induce replication stress and the reduction of BRCA1 pathway proteins leads to an increased frequency of chromatid-type aberrations. The notion that TTFields induces DNA replication stress can explain both the increase in DNA damage foci and the elevated frequency of chromatid-type aberrations. Ongoing studies by our group are seeking to better understand the molecular underpinning of this induced replication stress.

The reduced DNA DSB repair capacity seen in all cell lines when TTFields were applied post-IR is clearly linked to reduced cell survival (Figure 6 and Table 2). These data are consistent with findings reported recently by Kim *et al.*^21^ in which TTFields sensitized GBM cell lines to IR. In contrast to our methods, these authors applied TTFields prior to irradiation, whereas we first irradiated the cells and then immediately applied TTFields assuming that the chromosomal damage generated by IR would enhance the disruption of mitosis caused by TTFields exposure. Interestingly, both prior and post-TTFields treatment sensitize cells to IR, which could have an impact on treatment sequencing.

In conclusion, TTFields induce a global antiproliferative and cytotoxic effect on dividing cell populations; however, the relative sensitivity of cells to TTFields varies. These antitumor properties are due to multiple mechanisms, likely acting in concert, that would suggest TTFields should be utilized as an adjuvant modality with RT and other agents. Indeed, our data suggest by gaining additional insight into understanding the underlying molecular mechanisms governing TTFields' antitumor effects optimizing combinatorial strategies of TTFields, IR and other agents in preclinical models is appropriate. At this junction PARP inhibitors are particularly attractive. Lastly, whether specific molecular signatures as reported in this study will predict which patients will respond better to TTFields is worth exploring clinically where possible.

## Materials and methods

### Cell culture

Human NSCLC cell lines (H157, H4006, A549, H1299 and H1650) were purchased from American Tissue Culture Collection. All these cell lines were grown in RPMI medium^37, 38^ supplemented with 10% (v/v) fetal bovine serum (Atlanta Biologicals, Flowery Branch, GA, USA) and penicillin/streptavidin (final concentration 50 *μ*g/ml; Sigma-Aldrich, St. Louis, MO, USA). All cells were grown at 37 °C in a humidified incubator constantly supplied with 5% CO_2_.

### Tumor treating fields

We used the inovitro system (NovoCure Ltd, Haifa, Israel) to generate TTFields that use two pairs of electrodes printed perpendicularly on the outer walls of a Petri dish composed of high dielectric constant ceramic (lead magnesium niobate-lead titanite (PMN-PT)). The transducer arrays were connected to a sinusoidal waveform generator that generate low-intensity electric fields at the desired frequencies in the medium as summarized in Table 1. The orientation of the TTFields was switched 90^o^ every 1 s, thus covering the majority of the orientation axis of cell divisions, as previously described by Kirson *et al.*^15^ Plate temperature was maintained at 37 °C by placing the plates in a refrigerated incubator where the temperature was maintained at 19 °C to dissipate the heat generated by the inovitro system. The temperature was measured by 2 thermistors (Omega Engineering, Stamford, CT, USA) attached to the ceramic walls. All cell suspensions were grown on a cover slip inside the inovitro dish (NovoCure Ltd) and treated with TTFields for the times indicated in the figure legends.

### Cell growth assay

Human NSCLC (H157, H4006, A549, H1299 and H1650) cell lines were treated with different frequencies of TTFields indicated for 24, 48 and 72 h, and cell growth was counted using a Beckman coulter counter (Beckman Coulter Inc, Indianapolis, IN, USA) in triplicates for each sample. Growth curve graphs were drawn using the average cell number counted at each time point and the given TTFields frequency using GraphPad Prism V.6 (GraphPad Software Inc, La Jolla, CA, USA).

### Cell cycle analysis

Cells at specific times and treatments were harvested and fixed in 75% ice-cold ethanol at −20 °C for 24 h. Fixed cells were washed with PBS and incubated in 500 *μ*l of PI staining solution, that is, PBS containing 1 mg/ml RNAse A (Sigma-Aldrich), 0.05% triton X-100 and 30 *μ*g/ml of PI (Sigma-Aldrich) for 30 min at 37 °C. The cell cycle distribution was determined using a FACSCalibur system (BD Biosciences, San jose, CA, USA). More than 10 000 cells per sample were counted and the results were analyzed using FlowJo software v8.7.1 (Tree Star Inc, Ashland, OR, USA).

### Labeling and hybridization of RNA for gene expression analysis

Illumina Whole Genome HumanWG6 v4 Expression BeadChips (Illumina Inc, San Diego, CA, USA) were used. Each RNA sample (0.5 *μ*g) was amplified using the Illumina TotalPrep RNA amplification kit with biotin UTP (Enzo Life Sciences, Inc., Farmingdale, NY, USA) labeling. T7 oligo(dT) primers were used to generate single-stranded cDNA followed by a second-strand synthesis to generate double-stranded cDNA, which is then column-purified. *In vitro* transcription was done to synthesize biotin-labeled cRNA using T7 RNA polymerase. The cRNA was then column-purified and checked for size and yield using the Bio-Rad Experion system (Bio-Rad Laboratories, Hercules, CA, USA). cRNA (1.5* μ*g) was then hybridized for each array using standard Illumina protocols with streptavidin-Cy3 (Amersham Biosciences, Piscataway, NJ, USA) being used for detection. Slides were scanned on an Illumina Beadstation (Illumina Inc).

### Data processing and significance analysis of differential gene expression

Summarized expression values for each probe set were generated using BeadStudio 3.1 (Illumina Inc). The data were background-subtracted and quantile–quantile-normalized across samples using the MBCB algorithm.^39^ Normalized gene expression values were used to generate plots for comparisons. Analysis of differentially expressed genes in treated cell lines was performed using SAM. FDR<0.05 was considered to be statistically significant. Clustering analysis and heatmaps were generated using the Partek Genomic Suite software (Partek Incorporated, St. Louis, MO, USA). Gene ontology and pathway analysis was performed using IPA (QIAGEN, Redwood City, CA, USA).

### Immunoblotting

Laemmli sample buffer (4 × ; Bio-Rad Laboratories) was added to 30 *μ*g of each protein sample and the mixtures were boiled at 95 °C for 10 min. Protein mixtures were then loaded on 10% SDS-PAGE gel followed by transfer to PVDF membrane for 1 h at 90 V at 4 °C. The membrane was blocked with 5% fat-free milk in PBST for 1 h at room temperature and probed with anti *β*-actin (1:5000; Cell Signaling, Danvers, MA, USA), anti-BRCA1 (1:1000), anti-FANCD2 (1:2000) and anti-FANCA (1:500; Novus Biologicals LLC, Littleton, CO, USA) in PBST containing 2% bovine serum albumin (Thermo Fisher Scientific Inc, Bridgewater, NJ, USA) overnight at 4 °C. Membranes were washed with phosphate-buffered saline with 0.1% Tween-20 (PBST; 3 ×, 10 min, each) followed by incubation with secondary antibodies (1:5000) conjugated with horseradish peroxidase (GE Healthcare, Buckinghamshire, UK) for 1 h at room temperature. Membranes were developed using a chemiluminescence detection kit (Thermo Scientific, Rockford, IL, USA) on FluorChem M system (ProteinSimple, San Jose, CA, USA). Quantification was done using the ImageJ software (NIH, Bethesda, MD, USA) and normalized using the corresponding actin density.

### Immunofluorescence

Cells were seeded on glass coverslips and after treatment cells were washed and fixed with ice-cold methanol. The samples were blocked with 10% normal goat serum for 1 h and incubated with phospho-histone-*γ*-H2AX antibody (Ser139; Upstate Biotechnology, Temecula, CA, USA) and p53-binding protein 1 (53BP1) antibody (Cell Signaling). Samples were washed three times for 5 min in PBS, and then incubated with Alexa Fluor 488-conjugated anti-rabbit antibody and Alexa Fluor 555-conjugated anti-mouse antibody (Invitrogen, Carlsbad, CA, USA) for 1 h. Nuclei were counterstained with DAPI contained in Vecatshield mounting medium (Vector Laboratories Inc, Burlingame, CA, USA). The stained cells were then analyzed under a fluorescence microscope (Axio Imager M2, Carl Zeiss, Thornwood, NY, USA) with a × 63 objective (oil immersion, aperture 1.3) with five slices of z-stacks of 0.2 *μ*M thickness each. Quantitative image analysis of 40 nuclei from each experiment was performed using Cell module in Imaris software version 8.0 (Bitplane, Concord, MA, USA).

### Cytogenetic analyses

Preparation of metaphase chromosome spreads and cytogenetic analysis were performed as previously reported.^40^ Briefly, cultured cells were treated with 1 *μ*M colcemid solution (Thermo Scientific) for 3–4 h at 37 °C, trypsinized, incubated for 30 min in a hypotonic solution of 75 mM KCl solution and subsequently fixed with 3:1 methanol to acetic acid. Samples were then dropped on to glass slides and stained with either 5% Giemsa (Sigma-Aldrich) or prolong antifade gold reagent with DAPI (Life Technologies, Carlsbad, CA, USA) for scoring. The presence of chromosome-type aberrations (deletions, dicentric chromosomes and rings) and chromatid-type (gaps, breaks, deletions and radial chromosome arrangements) was detected under a microscope (Axio Imager M2, Carl Zeiss) and ~30 metaphase cells per treatment group were scored and averages displayed as the frequency of aberrations per cell.

### Radiation exposure and clonogenic cell survival

To study the effect of radiation sensitivity on NSCLC cells, exponentially growing cells were treated with IR using a Mark II ^137^Cs irradiator (J L Shepherd and Associates) at a dose rate of 3.47 Gy/min, followed by immediate application of TTFields for 24, 48 and 72 h. Cells were then re-seeded into 60 mm dishes and incubated for up to 2 weeks. Colonies containing 50 or more cells were considered viable. The data are presented as the mean±S.E.M. of three independent experiments. The radiosensitization effect of TTFields was evaluated according to The Highest Single Agent^41, 42, 43^ approach by calculating the CI as given below.


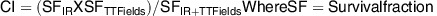


The combination effect was considered enhanced/synergistic when CI>1, additive when CI=1. Statistical significance for a positive effect was determined by the *P*-value of a two-way ANOVA multiple comparison statistical test comparing the combination (TTFields plus IR) to the single agent showing the greatest cell killing for a given dose and time after IR.

## Figures and Tables

**Table 1:** Standardization of frequencies in NSCLC cells and their genetic background

**Cell line**	**Standardized TTFields frequency (**kHz**)**	**% of growth inhibition at 72 h**	**P53 status**	**KRAS status**	**Doubling time (h)**
H157	100	72	Mutant	Wild type	36
H4006	150	69	SNP MS	Wild type	34
A549	200	48	Wild type	Mutant	22
H1299	100	32	Mutant	Wild type	20
H1650	100	21	Wild type	Wild type	26

List of cell lines used and their optimized frequency at which maximal growth inhibition was observed, average percentage of growth inhibition at the optimized frequency after 72 h of TTFields exposure, genetic background information and cell cycle-doubling time (*N*=3)

**Table 2:** Evaluation of radiosensitization effect of TTFields in combination with IR

**Cell line**	**Time point (h)**	**Combination index (CI) TTFields+2 Gy**	***P*-value**	**Combination index (CI) TTFields+4 Gy**	***P*-value**
H157	24	**1.48**	<0.0001	**2.23**	0.001
	48	**2.08**	<0.0001	**1.14**	0.003
	72	**1.15**	0.048	**1.18**	0.01
H4006	24	0.88	0.14	**1.88**	0.001
	48	**1.01**	<0.0001	**1.74**	0.003
	72	0.58	0.004	**1.01**	0.05
A549	24	**1.88**	<0.0001	1.50	0.12
	48	**1.14**	0.001	**2.36**	<0.0001
	72	0.86	0.14	**1.99**	0.0007
H1650	24	1.48	0.17	1.19	0.64
	48	**1.21**	<0.0001	0.90	0.21
	72	**1.35**	<0.0001	**1.47**	0.03
H1299	24	0.91	0.68	**3.32**	<0.0001
	48	0.79	0.09	**3.97**	<0.0001
	72	0.94	0.04	**2.42**	0.0003

Radiosensitization of TTFields was evaluated by the *Highest Single Agent* approach for combinations of 2 Gy+TTFields and 4 Gy+TTFields. Bold text denotes a statistically significant synergistic effect (CI>1 and *P*-value⩽0.05) for given time point post IR and a given cell line (*N*=3)

## References

[bib1] Siegel R, Desantis C, Jemal A. Colorectal cancer statistics, 2014. CA Cancer J Clin 2014; 64: 104–117. 24639052 10.3322/caac.21220

[bib2] Johnson DH. Setting the bar for therapeutic trials in non-small-cell lung cancer: how low can we go? J Clin Oncol 2014; 32: 1389–1391. 24687823 10.1200/JCO.2014.55.1929

[bib3] Johnson DH, Schiller JH, Bunn PA Jr.. Recent clinical advances in lung cancer management. J Clin Oncol 2014; 32: 973–982. 24567433 10.1200/JCO.2013.53.1228

[bib4] Morgensztern D, Goodgame B, Govindan R. Vaccines and immunotherapy for non-small cell lung cancer. J Thorac Oncol 2010; 5(12 Suppl 6): S463–S465. 21102240 10.1097/01.JTO.0000391367.63882.79

[bib5] Morgensztern D, Govindan R. Best of the month: a roundup of articles published in recent months. J Thorac Oncol 2010; 5: 1305–1307. 20661092 10.1097/JTO.0b013e3181ec809b

[bib6] Giladi M, Schneiderman RS, Porat Y, Munster M, Itzhaki A, Mordechovich D et al. Mitotic disruption and reduced clonogenicity of pancreatic cancer cells *in vitro* and *in vivo* by tumor treating fields. Pancreatology 2014; 14: 54–63. 24555979 10.1016/j.pan.2013.11.009

[bib7] Vymazal J, Wong ET. Response patterns of recurrent glioblastomas treated with tumor-treating fields. Semin Oncol 2014; 41(Suppl 6): S14–S24. 10.1053/j.seminoncol.2014.09.009 25213870

[bib8] Wong ET, Lok E, Swanson KD, Gautam S, Engelhard HH, Lieberman F et al. Response assessment of NovoTTF-100A versus best physician's choice chemotherapy in recurrent glioblastoma. Cancer Med 2014; 3: 592–602. 24574359 10.1002/cam4.210 PMC4101750

[bib9] Inui T, Amitani H, Kubo K, Kuchiike D, Uto Y, Nishikata T et al. Case report: a non-small cell lung cancer patient treated with GcMAF, sonodynamic therapy and tumor treating fields. Anticancer Res 2016; 36: 3767–3770. 27354652

[bib10] Davies AM, Weinberg U, Palti Y. Tumor treating fields: a new frontier in cancer therapy. Ann N Y Acad Sci 2013; 1291: 86–95. 23659608 10.1111/nyas.12112

[bib11] Gonzalez CF, Remcho VT. Harnessing dielectric forces for separations of cells, fine particles and macromolecules. J Chromatogr A 2005; 1079: 59–68. 16038291 10.1016/j.chroma.2005.03.070

[bib12] Stupp R, Taillibert S, Kanner AA, Kesari S, Steinberg DM, Toms SA et al. Maintenance therapy with tumor-treating fields plus temozolomide vs temozolomide alone for glioblastoma: a randomized clinical trial. JAMA 2015; 314: 2535–2543. 26670971 10.1001/jama.2015.16669

[bib13] Giladi M, Schneiderman RS, Voloshin T, Porat Y, Munster M, Blat R et al. Mitotic spindle disruption by alternating electric fields leads to improper chromosome segregation and mitotic catastrophe in cancer cells. Sci Rep 2015; 5: 18046. 26658786 10.1038/srep18046 PMC4676010

[bib14] Kirson ED, Dbaly V, Tovarys F, Vymazal J, Soustiel JF, Itzhaki A et al. Alternating electric fields arrest cell proliferation in animal tumor models and human brain tumors. Proc Natl Acad Sci USA 2007; 104: 10152–10157. 17551011 10.1073/pnas.0702916104 PMC1886002

[bib15] Kirson ED, Gurvich Z, Schneiderman R, Dekel E, Itzhaki A, Wasserman Y et al. Disruption of cancer cell replication by alternating electric fields. Cancer Res 2004; 64: 3288–3295. 15126372 10.1158/0008-5472.can-04-0083

[bib16] Gera N, Yang A, Holtzman TS, Lee SX, Wong ET, Swanson KD. Tumor treating fields perturb the localization of septins and cause aberrant mitotic exit. PLoS ONE 2015; 10: e0125269. 26010837 10.1371/journal.pone.0125269 PMC4444126

[bib17] Schneiderman RS, Shmueli E, Kirson ED, Palti Y. TTFields alone and in combination with chemotherapeutic agents effectively reduce the viability of MDR cell sub-lines that over-express ABC transporters. BMC Cancer 2010; 10: 229. 20492723 10.1186/1471-2407-10-229 PMC2893108

[bib18] Giladi M, Weinberg U, Schneiderman RS, Porat Y, Munster M, Voloshin T et al. Alternating electric fields (tumor-treating fields therapy) can improve chemotherapy treatment efficacy in non-small cell lung cancer both *in vitro* and *in vivo*. Semin Oncol 2014; 41(Suppl 6): S35–S41. 25213867 10.1053/j.seminoncol.2014.09.006

[bib19] Kirson ED, Schneiderman RS, Dbaly V, Tovarys F, Vymazal J, Itzhaki A et al. Chemotherapeutic treatment efficacy and sensitivity are increased by adjuvant alternating electric fields (TTFields). BMC Med Phys 2009; 9: 1. 19133110 10.1186/1756-6649-9-1 PMC2647898

[bib20] Kirson ED, Giladi M, Gurvich Z, Itzhaki A, Mordechovich D, Schneiderman RS et al. Alternating electric fields (TTFields) inhibit metastatic spread of solid tumors to the lungs. Clin Exp Metastasis 2009; 26: 633–640. 19387848 10.1007/s10585-009-9262-y PMC2776150

[bib21] Kim EH, Kim YJ, Song HS, Jeong YK, Lee JY, Sung J et al. Biological effect of an alternating electric field on cell proliferation and synergistic antimitotic effect in combination with ionizing radiation. Oncotarget 2016; 7: 62267–62279. 27556699 10.18632/oncotarget.11407 PMC5308725

[bib22] Voloshin T, Munster M, Blatt R, Shteingauz A, Roberts PC, Schmelz EM et al. Alternating electric fields (TTFields) in combination with paclitaxel are therapeutically effective against ovarian cancer cells *in vitro* and *in vivo*. Int J Cancer 2016; 139: 2850–2858. 27561100 10.1002/ijc.30406 PMC5095795

[bib23] Roy R, Chun J, Powell SN. BRCA1 and BRCA2: different roles in a common pathway of genome protection. Nat Rev Cancer 2011; 12: 68–78. 22193408 10.1038/nrc3181 PMC4972490

[bib24] McPherson JP, Hande MP, Poonepalli A, Lemmers B, Zablocki E, Migon E et al. A role for Brca1 in chromosome end maintenance. Hum Mol Genet 2006; 15: 831–838. 16446310 10.1093/hmg/ddl002

[bib25] Patel KJ, Yu VP, Lee H, Corcoran A, Thistlethwaite FC, Evans MJ et al. Involvement of Brca2 in DNA repair. Mol Cell 1998; 1: 347–357. 9660919 10.1016/s1097-2765(00)80035-0

[bib26] Cavanagh H, Rogers KM. The role of BRCA1 and BRCA2 mutations in prostate, pancreatic and stomach cancers. Hered Cancer Clin Pract 2015; 13: 16. 26236408 10.1186/s13053-015-0038-x PMC4521499

[bib27] Kan C, Zhang J. BRCA1 mutation: a predictive marker for radiation therapy? Int J Radiat Oncol Biol Phys 2015; 93: 281–293. 26383678 10.1016/j.ijrobp.2015.05.037 PMC4576355

[bib28] Kaelin WG Jr.. The concept of synthetic lethality in the context of anticancer therapy. Nat Rev Cancer 2005; 5: 689–698. 16110319 10.1038/nrc1691

[bib29] Turner N, Tutt A, Ashworth A. Hallmarks of 'BRCAness' in sporadic cancers. Nat Rev Cancer 2004; 4: 814–819. 15510162 10.1038/nrc1457

[bib30] Schultz LB, Chehab NH, Malikzay A, Halazonetis TD. p53 binding protein 1 (53BP1) is an early participant in the cellular response to DNA double-strand breaks. J Cell Biol 2000; 151: 1381–1390. 11134068 10.1083/jcb.151.7.1381 PMC2150674

[bib31] Huyen Y, Zgheib O, Ditullio RA Jr., Gorgoulis VG, Zacharatos P, Petty TJ et al. Methylated lysine 79 of histone H3 targets 53BP1 to DNA double-strand breaks. Nature 2004; 432: 406–411. 15525939 10.1038/nature03114

[bib32] Venere M, Snyder A, Zgheib O, Halazonetis TD. Phosphorylation of ATR-interacting protein on Ser239 mediates an interaction with breast-ovarian cancer susceptibility 1 and checkpoint function. Cancer Res 2007; 67: 6100–6105. 17616665 10.1158/0008-5472.CAN-07-0369

[bib33] Cescutti R, Negrini S, Kohzaki M, Halazonetis TD. TopBP1 functions with 53BP1 in the G1 DNA damage checkpoint. EMBO J 2010; 29: 3723–3732. 20871591 10.1038/emboj.2010.238 PMC2982761

[bib34] Sirbu BM, Couch FB, Feigerle JT, Bhaskara S, Hiebert SW, Cortez D. Analysis of protein dynamics at active, stalled, and collapsed replication forks. Genes Dev 2011; 25: 1320–1327. 21685366 10.1101/gad.2053211 PMC3127432

[bib35] Chan KL, Palmai-Pallag T, Ying S, Hickson ID. Replication stress induces sister-chromatid bridging at fragile site loci in mitosis. Nat Cell Biol 2009; 11: 753–760. 19465922 10.1038/ncb1882

[bib36] Su F, Bhattacharya S, Abdisalaam S, Mukherjee S, Yajima H, Yang Y et al. Replication stress induced site-specific phosphorylation targets WRN to the ubiquitin-proteasome pathway. Oncotarget 2016; 7: 46–65. 26695548 10.18632/oncotarget.6659 PMC4807982

[bib37] Das AK, Sato M, Story MD, Peyton M, Graves R, Redpath S et al. Non-small-cell lung cancers with kinase domain mutations in the epidermal growth factor receptor are sensitive to ionizing radiation. Cancer Res 2006; 66: 9601–9608. 17018617 10.1158/0008-5472.CAN-06-2627

[bib38] Phillips RJ, Mestas J, Gharaee-Kermani M, Burdick MD, Sica A, Belperio JA et al. Epidermal growth factor and hypoxia-induced expression of CXC chemokine receptor 4 on non-small cell lung cancer cells is regulated by the phosphatidylinositol 3-kinase/PTEN/AKT/mammalian target of rapamycin signaling pathway and activation of hypoxia inducible factor-1alpha. J Biol Chem 2005; 280: 22473–22481. 15802268 10.1074/jbc.M500963200

[bib39] Ding LH, Xie Y, Park S, Xiao G, Story MD. Enhanced identification and biological validation of differential gene expression via Illumina whole-genome expression arrays through the use of the model-based background correction methodology. Nucleic Acids Res 2008; 36: e58. 18450815 10.1093/nar/gkn234 PMC2425463

[bib40] Sishc BJ, Nelson CB, McKenna MJ, Battaglia CL, Herndon A, Idate R et al. Telomeres and telomerase in the radiation response: implications for instability, reprograming, and carcinogenesis. Front Oncol 2015; 5: 257. 26636039 10.3389/fonc.2015.00257 PMC4656829

[bib41] Foucquier J, Guedj M. Analysis of drug combinations: current methodological landscape. Pharmacol Res Perspect 2015; 3: e00149. 26171228 10.1002/prp2.149 PMC4492765

[bib42] Geary N. Understanding synergy. Am J Physiol Endocrinol Metab 2013; 304: E237–E253. 23211518 10.1152/ajpendo.00308.2012

[bib43] Lehar J, Zimmermann GR, Krueger AS, Molnar RA, Ledell JT, Heilbut AM et al. Chemical combination effects predict connectivity in biological systems. Mol Syst Biol 2007; 3: 80. 17332758 10.1038/msb4100116 PMC1828746

