# Supplementary material for: Tumor-treating fields elicit a conditional vulnerability to ionizing radiation via the downregulation of BRCA1 signaling and reduced DNA double-strand break repair capacity in non-small cell lung cancer cell lines
Source: Cell Death Dis. 2017 Mar 30;8(3):e2711–. doi: 10.1038/cddis.2017.136 (PMC5386539; doi:10.1038/cddis.2017.136)
Supplement: Supplementary Material [file cddis2017136x1.doc]

**Supplementary Figures:**

**Supplementary Figure 1: Standardization of optimal frequency for maximal cell growth inhibition in panel of NSCLC cell lines:** Cell growth was plotted against control for each cell line at different given frequencies in order to optimize the best frequency which shows maximal cell growth inhibition effect for each cell line.

**Supplementary Figure 2: Representative images of cell cycle analysis histograms:** Cell cycle analysis was carried out using PI staining at 24h, 48h and 72h time points and representative histograms are shown here indicating TTFields induced G2/M enrichment over time.

**Supplementary Figure 3: Microarray analysis strategy to understand the underlying mechanism of TTFields action.**  NSCLC cell lines were divided into two groups based on TTField response. More responsive (H4006 and H157): Less responsive (A549, H1650 and H1299). Cell lines and different time points taken for micro array analysis are outlined here.

**Supplementary Figure 4: Down regulation of BRCA1 pathway genes upon TTFs treatment.** Expression of BRCA1 pathway genes given for BRCA1, FANCE, FANCC, FANCB, FANCA and RFC3 all of which were down-regulated with increasing TTFs exposure time.

**Supplementary Figure 5: Representative immunofluorescence images of γ-H2AX and 53 BP1 foci.** Representative immunofluorescence images of γ-H2AX and 53 BP1 foci for different conditions and time points are shown here highlighting accumulation of γ-H2AX and 53 BP1 foci over time in TTFields and IR+TTFields conditions compared to their respective controls.

**Supplementary Figure 6: Representative images of chromosomal spreads.** Representative metaphase exposed to TTF for 48 hours demonstrating both a chromatid type chromatid-chromatid fusion event (red circle) and a chromosome type dicentric chromosomal aberration (green circle).
